# *In vitro* experiments of *Pediculus humanus capitis* (*Phthiraptera*: *Pediculidae*) resistance to permethrin and 6-paradol in East Jakarta: Detoxification enzyme activity and electron microscopic changes in lice

**DOI:** 10.14202/vetworld.2021.3065-3075

**Published:** 2021-11-30

**Authors:** Rizal Subahar, Lisawati Susanto, Rachmanin Aidilla, Annisa Putri Aulia, Yulhasri Yulhasri, Rawina Winita, Nadar S. Lubis, Ika Puspa Sari

**Affiliations:** 1 Department of Parasitology, Faculty of Medicine, University of Indonesia, Jl. Salemba 6, Jakarta 10430, Indonesia; 2 Medical Doctor Program, Faculty of Medicine, University of Indonesia, Jl. Salemba 4, Jakarta 10430, Indonesia; 3 Department of Biochemistry and Molecular Biology, Faculty of Medicine, University of Indonesia, Jln. Salemba Raya 4, Jakarta 10430, Indonesia

**Keywords:** 6-paradol, detoxifying enzyme, electron microscopy, *Pediculus humanus capitis*, permethrin, resistance

## Abstract

**Background and Aim:**

*Pediculus humanus capitis*, the human head louse, remains a global health problem. This study evaluated the resistance of head lice to permethrin and 6-paradol mediated by *in vitro* detoxification enzyme activity experiments and to describe physical changes in the lice using scanning electron microscopy (SEM).

**Materials and Methods:**

The adult stages of *P. h. capitis* were collected from patients exposed to 1% permethrin and three different concentrations of 6-paradol (0.00005%, 0.0001%, and 0.00015%) using a filter paper diffusion bioassay. Healthy *P. h. capitis* adults served as the control. The *in vitro* bioassays were conducted after 10, 20, 30, and 60 min of exposure. The activities of acetylcholinesterase (AChE), glutathione S-transferase (GST), and oxidase were analyzed. Physical changes in the lice were analyzed using SEM.

**Results:**

Permethrin and 6-paradol exhibited low toxicity against the lice. At 60 min, 1% permethrin had killed 36.7% of the lice present, while 6-paradol had killed 66.7-86.7%. Permethrin induced significantly elevated AChE, GST, and oxidase activity; 6-paradol also caused significantly elevated AChE, GST, and oxidase activity. Permethrin did not cause any ultrastructural morphological changes on the lice, while 6-paradol severely damaged the head, thorax, respiratory spiracles, and abdomen of the dead lice.

**Conclusion:**

This *in vitro* experimental of *P. h. capitis* is the first study to report *P. h. capitis* in East Jakarta shows complete resistance to permethrin and 6-paradol, and to describe the associated increase in AChE, GST, and oxidase activity. It was observed that 6-paradol severely damaged the head, thorax, respiratory spiracles, and abdomen of the dead lice.

## Introduction

Pediculosis is the infestation of the head with hematophagous lice *Pediculus humanus capitis* De Geer (*Phthiraptera*: *Pediculidae*), an obligate ectoparasite [1-3]. Pediculosis remains a global health problem and commonly affects children between 6 and 12 years of age [4,5]. The previousstudy has reported that the prevalence rates of pediculosis ranged from 13.3 to 49% in children aged 6-13 years in rural areas [1]. In Turkey, the prevalence range in children aged 6-11 years was 3.96-27.2%, and the infestation rate among girls was 3.4 times higher than that among boys [6]. The prevalence rates have been shown to be 67.3% in primary school girls in a low-income area in the southeast of Iran [7], 49% in children between the ages of ten and 12 in Malaysia [8], and 83% in rural populations in Mali [9]. Many negative effects of pediculosis have been reported, including social embarrassment, isolation, parental anxiety, peer criticism, and difficulty for school authorities [2,5]. Therefore, pediculosis has drawn the attention of researchers. Some have studied the biological aspects of lice concerning resistance to commonly used pediculicides [2,10].

The primary treatment for head lice involves the use of topical chemical drugs, which include a wide variety of neurotoxic synthetic insecticides, such as dichloro diphenyl tricholoroethane (DDT), lindane, malathion, carbaryl, permethrin (1%), and d-phenothrin [10,11]. However, these pediculicidal agents’ frequent and intensive use has caused lice to develop resistance to the drugs. In addition, multiple treatments for pediculosis, including overdoses, raise serious human health concerns [4]. A previous study [10] reported that pediculicide-resistant human lice corresponded to voltage-gated sodium channel (VGSC) α-subunit gene known as knockdown resistance (*kdr*), allowing pediculicides to kill the lice more slowly. These *kdr* mutations induce insensitivity of the nervous systems of insects to pediculides, such as DDT, organophosphates, and pyrethroids. Three *kdr*-type point mutations, MB15l, T917l, and L920F, have been found in permethrin- and DDT-resistant human lice [1,10].

Hodgdon *et al*. [12] reported that *kdr-*type point mutations are found worldwide. Uruguay, the UK, and Australia exhibited *kdr* allele frequencies of 100%, while the USA, Argentina, Brazil, Denmark, the Czech Republic, Egypt, and Israel exhibited *kdr* allele frequencies between 11% and 97%. Regarding primary school children in Thailand, *kdr* T9171 and L920F point mutations were discovered in permethrin-resistant head lice [13]. In Honduras [14], Chile [15], and Madagascar [16], the T917I *kd*r allele was found in head lice resistant to pyrethroid insecticides. However, in Senegal, it has been reported that the presence of a point mutation of GluCI is associated with ivermectin-resistant head lice [17]. In Iran, six novel point mutations, namely, H813P (located in IIS1-2 extracellular loop), I927F, L928A, R929V, L930M, and L932M (located in IIS5) in the VGSC α-subunit gene were found in head lice resistant to pyrethroid insecticides [18]. In Mexico, a new point mutation, T929I, was found in head lice resistant to insecticides, especially pyrethroids [19]. These *kdr-*type point mutations are induced by frequent and inadequate doses of pediculicidal agents, rendering alternative topical drugs necessary to treat these resistant human lice [1].

The resistant human lice mechanisms are associated with *kdr*-type mutations and with metabolic detoxi- fying enzyme systems, such as monooxygenases, esterases, and glutathione S-transferases (GST) [20]. Hemingway *et al*. [21] demonstrated increased GST and monooxygenase activity in head lice from Israel associated with resistance to DDT and permethrin. Brogdon [22] showed that individually resistant insects were associated with elevated detoxifying enzyme activity. These detoxifying enzyme systems are involved in permethrin-resistant lice mechanism, so that the lice survive under the pressures of pediculicidal agents.

So far, no studies have analyzed the resistance of human lice to permethrin in Jakarta, Indonesia’s capital city. Therefore, this study examined human lice from crowded urban areas in East Jakarta. The study focused on detecting the resistance of human lice to permethrin (1%) using *in vitro* experiments to select effective management strategies [10,12]. The mechanism of permethrin-resistant lice was examined concerning the detoxifying enzymes acetylcholinesterase (AChE), GST, and oxidase. Furthermore, scanning electron microscopy (SEM) was used to examine physical changes in the lice after *in vitro* treatment with permethrin compared with 6-paradol. For permethrin-resistant lice, this study used 6-paradol [1(4-hydroxy-methoxyphenyl)-3decen-one], a bioactive plant compound, to investigate the similar mechanisms of 6-paradol associated with detoxifying enzyme systems to kill lice in an *in vitro* setting. This study used 6-paradol because human lice develop resistance to chemical pediculicides, such as DDT, malathion, carbaryl, and lindane [1,10]. To the best of our knowledge, this is the first *in vitro* study to evaluate the resistance of human head lice from East Jakarta to permethrin and 6-paradol mediated by metabolic enzyme systems and to describe any changes in the lice as seen by SEM.

The compound 6-paradol is derived from 6-shogaol [1(4-hydroxy-methoxyphenyl)-4decen-one] through a biotransformation process [23,24]. The 6-shogaol comes from *Zingiber officinale* Roscoe (ginger), Zingiberaceae family. Pharmacologically, 6-shogaol and 6-paradol exhibit neuroprotective effects in a mouse model of multiple sclerosis; they exerted anti-neuroinflammatory and anti-oxidative activity in the central nervous system [25].

In addition, 6-paradol of *Z. officinale* origin has many target sites of detoxifying enzyme systems; therefore, this study aimed to evaluate the resistance of head lice to permethrin and 6-paradol mediated by *in vitr*o detoxification enzyme activity experiments and to describe physical changes in the lice using SEM.

## Materials and Methods

### Ethical approval

The study has passed the ethical approval of the Research Module Management Team of The Medical Faculty, University of Indonesia, Indonesia (KET-019/UN2.F1.DI.2/PDP.01/Riset-2/2021).

### Study period and location

**Figure-1:**
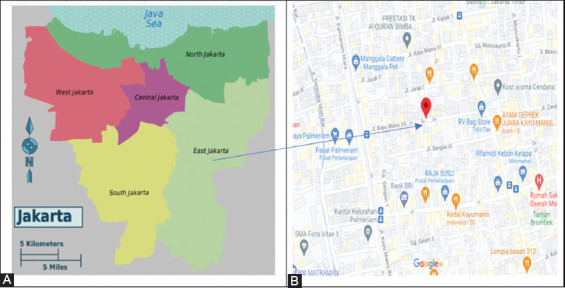
Study site of head lice in East Jakarta. Jakarta city map (A), and head lice sampling location map (B). [Image sources: (a) https://commons.wikimedia.org/wiki/File:Jakarta_Wikivoyage_Map.svg; (b) https://www.google.com/maps].

The study was conducted from January 2020 to February 2021. The study site was Jln. Kayu Manis VII RT 08 RW 07, Kayu Manis Subdistrict, Matraman District, East Jakarta (Figure-1). The samples were processed at Laboartory of Department of Parasitology, Faculty of Medicine, University of Indonesia.

### 6-paradol

The 6-paradol (catalog number S5551) was purchased from the manufacturer, P.T.Beatrix Indonesia, Jakarta, Indonesia. All of the larval bioassays used a 0.0025% stock solution of 6-paradol. Dimethyl sulfoxide was used as a solvent to dissolve 6-paradol. A series of concentrations of 6-paradol was used (0.00005%, 0.0001%, and 0.00015%) in triplicate.

### 1% permethrin

PediTox lotion containing 1% permethrin (PT Combiphar, No. Reg. DTL. 13041329411, Jakarta, Indonesia) was purchased in a local market in Central Jakarta, Indonesia.

### Collection of head lice

This study used the common method to collect head lice as previously described [26,27]. Briefly, the adult head lice were obtained from a population of children between the ages of 10 and 12 in East Jakarta, Indonesia, with the approval of their guardians. Lice were collected using a clean plastic louse comb, and the lice were carefully removed from the plastic comb teeth. After combing, the lice were placed into plastic boxes [26]. The subjects had not been treated with any pediculicide solution for at least the preceding month. As per the methods reported by Jadhav *et al*. [28], the head lice used in this study were protected from sunlight and heat, and the *in vitro* test was initiated within 1 h after collection.

### Filter paper diffusion bioassay

The filter paper diffusion method was used in this bioassay as previously described and modi- fied [26,28]. One milliliter of the test sample (permethrin or 6-paradol) was distributed evenly over a filter paper (4.5-cm diameter) held in the lower half of the glass Petri dish (5-cm diameter). After 10 min, ten head lice were placed carefully on the filter paper with the test sample with the help of a “0” fine hair-brush. The study used three replications. The number of head lice used for the treatment with permethrin was 30, 6-paradol was 90 (30 head lice for a concentration of 0.00005%, 30 head lice for a concentration of 0.0001%, and 30 head lice for a concentration of 0.00015%), and control was 30. Hence, the total head lice were 150. The petri dish was covered with a lid during the test. Observations of dead-head lice (% mortality) were recorded at 10, 20, 30, and 60 min post introduction of lice on filter paper impregnated with the test sample. The study used permethrin (1%), 6-paradol (0.00005 %, 0.0001%, and 0.00015%), and control (distilled water) were tested for pediculicidal activity.

After the bioassay was completed, 27 head lice treated with permethrin were used to analyze AChE, GST, and oxidase activity, and the remaining three head lice were used for SEM examination. To check the action of the enzyme, the same sample of head lice was used. The same was done in the treatment involving 6-paradol and control. In the 6-paradol group, 87 head lice were used to examine AChE, GST, and oxidase activity, and the remaining three head lice were used for SEM examination. In the control group, 30 head lice were used to examine the action of the enzyme.

### AChE, GST, and oxidase activity

This study analyzed the activity of AChE, GST, and oxidase using the same sample, namely, 30 head lice from the control group, 27 lice from the permethrin group, and 87 from the 6-paradol group. Eighty-seven head lice from the 6-paradol group consisted of 30 lice at a concentration of 0.00005%, 30 lice at a concentration of 0.0001%, and 27 lice at a concentration of 0.00015%. In this study, AChE, GST, and oxidase activities were conducted using the Centers for Disease Control and Prevention method [22]. The unit of enzyme activity is absorbance (Abs) per minute (min) or Abs/min [29].

In the AChE activity, the lice were homogenized with 1000-μL 0.25-M KPO_4_ (pH 7.2). At room temperature, 100-μL aliquots of test sample homogenate were loaded into enzyme-linked immunosorbent assay (ELISA) microplate wells in triplicate. Similarly, 100-μL positive and negative controls were added to microplate wells. Acetylcholine iodide and dithiobis (2-nitrobenzoic acid) were each added at a volume of 100-μL into the wells containing the test sample, posi- tive and negative controls, respectively. The plates were read immediately (T_0_) using an ELISA reader at 414-nm and read again at 10 min (T_10_). The unit of AChE activity was Abs 414-nm/min [22,29]. ELISA reader (ThermoFisher Scientific, Multiskan™ FC Microplate Photometer, Catalog number 51119000, type 300, Finland).

In the GST activity, the lice were homogenized with 1000-μL 0.25-M KPO_4_ (pH 7.2). At room temperature, 100-μL aliquots of test sample homogenate were loaded into ELISA microplate wells in triplicate. Similarly, 100-μL positive and negative controls were added to microplate wells. Next, 100-μL reduced glutathione solution (Sigma G4251, China) and 100-μL 1-chloro-2,4’-dinitrobenzene were added to each well containing a test sample. The plates were read immediately (T_0_) with an ELISA reader at 340-nm and read again at 5 min (T_5_). The unit of GST activity was Abs 340-nm/min [22,26].

The lice were homogenized with 1000-μL 0.25 M KPO_4_ (pH 7.2) in the oxidase activity. The study used positive controls (i) 1:55 (22-μL cytochrome stock, 1.2-mL KPO_4_ buffer) and (ii) 1:110 (11-μL cytochrome stock, 1.2-mL KPO_4_ buffer). At room temperature, 100-μL aliquots of the test sample homogenate were loaded into ELISA microplate wells in triplicate. Next, 100-μL KPO_4_ was added to the negative and positive sample wells. Afterward, 100-μL cytochrome-C-positive control (cytochrome-C bovine heart) and 200-μL 3,3’,5,5’-Tetramethyl-Benzidine Dihydronchloride solution were added to each well. One drop of 3% hydrogen peroxide (H_2_O_2_) was added to each well and incubated for 5 min. The plates were read immediately (T_0_) with an ELISA reader at 620-nm. The unit of oxidase activity was Abs 414-nm/min [22,29]

### SEM study

This study evaluated the electron microscopic changes in the lice exposed to permethrin and 6-paradol according to the protocol described by Nunes *et al*. [30]. Briefly, the phases of the process are as follows: The lice were pre-fixed in 2.5% glutaraldehyde at 4°C for 6 h and then fixed in 1% osmium tetroxide for 2 h and rinsed in 0.1-M phosphate buffer (pH 7.2). Then, the samples were dehydrated in an acetone series in ascending order (30%, 50%, 70%, 80%, 90%, and 100% v/v) for 15 min each and in absolute acetone twice for 20 min, then embedded in epoxy resin (Beijing Daji Keyi Technology Co., Ltd., Beijing, China). Specimens were mounted on stubs with double-sided carbon contact tape and observed using a JEOL JSM-639OLA analytical scanning electron microscope. A low-vacuum chamber pressure of 150 Pa was used. Images of the specimens were taken at 3024×2304 pixels and scanned at a speed of 12 min 54 s.

### Statistical analysis

All statistical analyses, including the lice mortality rate, probit analysis, and detoxifying enzyme activity, were conducted using Statistical Package for the Social Sciences version 20.0. The louse mortality rate (%) was calculated using the following formula:


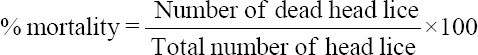


Lethal concentration of 50% (LC_50_) and lethal time of 50% (LT_50_) values were calculated by probit analysis. AChE, GST, and oxidase activities were calculated according to the formula below [22,29];


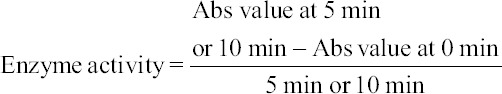


The permethrin-resistant lice were distinguished by the poor efficacy of permethrin in the *in vitro* experiments and increases in all detoxifying enzyme activities [22,31]. The 6-paradol-resistant lice were similarly identified. Twenty-seven head lice from the permethrin group and 87 head lice from the 6-paradol group (30 head lice from a concentration of 0.00005%, 30 head lice from a concentration of 0.0001%, and 27 head lice from a concentration of 0.00015%) were used to test t for paired samples. Paired samples t-test tests two paired samples, whether they have significantly different means or not. The two paired samples are the absorbance values of AChE, GST, and oxidase at 0 min to 5 or 10 min. All samples were normally distributed. Differences were considered statistically significant at p<0.05 [32]

## Results

### Movement of the head lice

**Figure-2:**
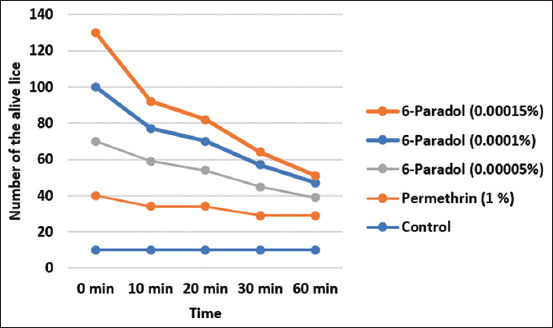
Numbers of live lice after *in vitro* exposure to permethrin and 6-paradol at 0, 10, 20, 30, and 60 min.

At 0 min, all lice in the control, permethrin, and 6-paradol groups moved actively. In the control group, all lice moved actively at all-time points (100%, 10/10). In the permethrin group, at ten and 20 min, as much as 80% (24/30) of the lice were still actively moving or alive. In contrast, at 30 and 60 min, only 63.3% (19/30) of the lice were still alive. In the 6-paradol group, live lice differed according to the concentrations of 6-paradol. At 60 min, the live lice comprised 33.3% (10/30), 26.7% (8/30), and 13.3% (4/30) in 0.00005%, 0.0001%, and 0.00015% 6-paradol, respectively. Figure-2 indicates the numbers of live lice after *in vitro* exposure to permethrin and 6-paradol at all-time points.

### Efficacy of permethrin and 6-paradol

**Figure-3:**
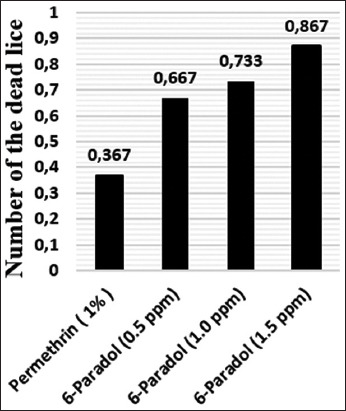
The efficacy of permethrin and 6-paradol against lice *in vitro*.

Table-1 indicates the head lice mortality rate after *in vitro* treatment with 1% permethrin and 6-paradol. At all-time points, the healthy control samples showed no dead lice (0.0%). For the permethrin group, at 60 min, the mortality rate was 36.7% (11/30). In contrast, at 60 min, 6-paradol killed 66.7%, 73.3%, and 86.7% of the lice at 0.00005%, 0.0001%, and 0.00015%, respectively. Overall, permethrin and 6-paradol exhibited low efficacy (Figure-3). The LT_50_ and LT_90_ values for permethrin and 6-paradol are indicated in Table-2. For permethrin, the values were recorded at 37.666 and 354.670 min, respectively. In contrast, for 6-paradol, the values varied according to the concentration. The highest concentration of 6-paradol (0.00015%) exhibited LT_50_ and LT_90_ values of 9.926 and 76.160 min, respectively. The lower concentrations of 6-paradol, 0.00005%, and 0.0001% had higher LT_50_ and LT_90_ values than the high concentration. Overall, *in vitro* treatment with permethrin killed lice more slowly than that of 6-paradol.

**Table 1:** Mortality rates of head lice after exposure to permethrin and 6-paradol

Treatment	n	10 min		20 min		30 min		60 min	
			
		Dead	%	Dead	%	Dead	%	Dead	%
Control	30	0	0	0	0	0	0	0	0
Permethrin (1%)	30	6	20	6	20	11	36.7	11	36.7
6-paradol									
0.00005%	30	5	16.7	10	33.3	14	46.7	20	66.7
0.0001%	30	2	6.7	16	53.3	18	60	22	73.3
0.00015%	30	15	50	20	66.7	23	76.7	26	86.7

N=Number of the lice

**Table 2:** The LT of permethrin and 6-paradol

Treatment	Z	SE	p-value	95% CI		λ^2^	p-value	LT_50_ (min)	LT_90_ (min)

				Lower	Upper				
Permethrin 1%	2.597	0.507	0.009	0.323	2.309	37.490	0.000	37.666 (-)	354.670 (-)
6-paradol									
0.00005%	4.391	0.892	0.000	2.168	5.662	16.990	0.000	17.433 (10.269–30.456)	37.042 (24.045–494.260)
0.0001%	4.597	1.147	0.000	3.024	7.519	17.54	0.000	18.338 (15.120–23.310)	32.097 (24.818–55.796)
0.00015%	3.177	0.456	0.001	0.555	2.341	7.844	0.646	9.926 (2.387–15.481)	76.160 (43.377–617.935)

SE=Standard error, Sig=Significant, CI=Confidence interval, LT=Lethal time

### Detoxifying enzyme activity

**Figure-4:**
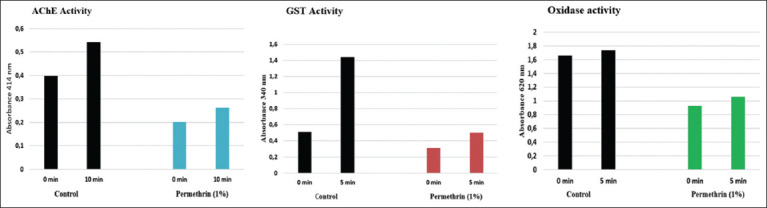
In the permethrin group, AChE, GST, and oxidase activity increased from 0 min to 5 min or 10 min. AChE (absorbance 414 nm), GST (absorbance 340 nm), oxidase (absorbance 620 nm). Control group is black, permethrin group is blue (AChE), red (GST), and green (oxidase). AChE=Acetylcholinesterase, GST=Glutathione S-transferase.

**Figure-5:**
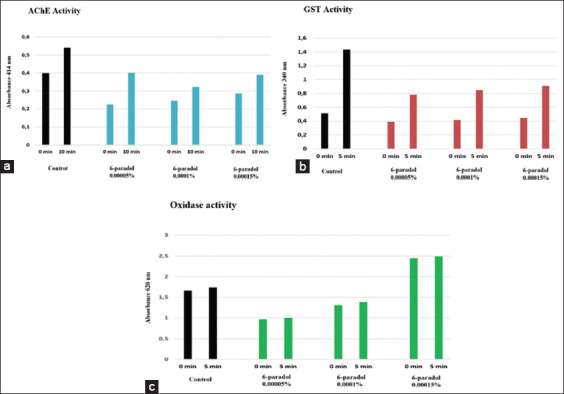
In the 6-paradol group, all concentrations of 6-paradol caused an increase in AChE, GST, and oxidase activity from 0 min to 5 min or 10 min. AChE (a) (absorbance 414 nm), (b) GST (absorbance 340 nm), (c) oxidase (absorbance 620 nm). AChE=Acetylcholinesterase, GST=Glutathione S-transferase.

This study used 30 head lice from the control group, 27 from the permethrin group, and 87 from the 6-paradol group to examine AChE, GST, and oxidase activity. All head lice (100%, 30/30) had increased AChE, GST, and oxidase activity in the control group. The mean Abs value for AChE at 0 min was 0.398±0.016 and increased for 10 min then to 0.541±0.020 (Figures-4 and 5). In GST, the mean abs at 0 min were 0.511±0.007 and increased to 1438±0.005 in 5 min later (Figures-4 and 5). In oxidase, the mean Abs value at 0 min was 1.661±0.001 and increased to 1.733±0.001 (Figures-4 and 5) 5 min later. The percentage increase in Abs values from AChE (0-10 min) was 36.1±6.74%, GST (0-5 min) was 181.3±3.70%, and oxidase (0-5 min) was 4.4±0.04%. In addition, the average increase in AChE, GST, and oxidase activity (Abs/min) was 0.014±0.002, 0.093±0.001, and 0.007±0.0 Abs/min, respectively.

**Figure-6:**
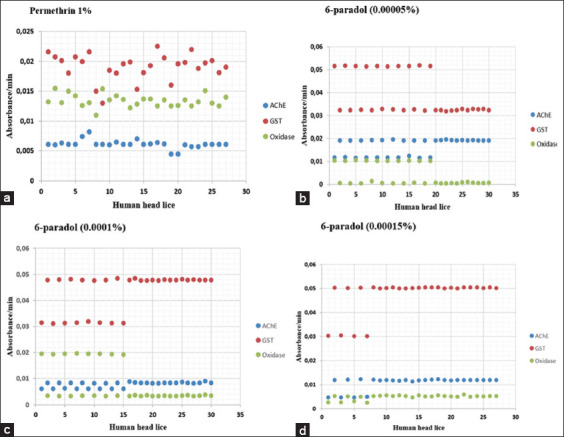
(a and b) AChE, GST, and oxidase activity of each individual head louse exposed to permethrin (1%) and 6-paradol (0.00005%). AChE (blue), GST (red), oxidase (green). The number of lice consisted of 27 head lice from permethrin group and 30 head lice from 6-paradol (0.00005%) group. (c and d) AChE, GST, and oxidase activity of each individual head louse exposed to 6-paradol (0.0001%) and 6-paradol (0.00015%). AChE (blue), GST (red), oxidase (green). The number of lice consisted of 30 head lice from 6-paradol (0.0001%) group and 27 head lice from 6-paradol (0.00015%) group. AChE=Acetylcholinesterase, GST=Glutathione S-transferase.

In the permethrin group, all head lice (100%, 27/27) exhibited an increase in the Abs values of AChE, GST, and oxidase from 0 to 5 min or 10 min (Figure-4). There was an increase in the mean value of Abs AChE (0-10 min) by 30.6±3.83%, GST (0-5 min) by 61.7±7.14%, and oxidase (0-5 min) by 14.5±1.2%. In addition, this study found a fluctuating increase in AChE, GST, and oxidase (Abs/min) activity in each head lice (Figure-6a). The average increase in AChE, GST, and oxidase activity (Abs/min) increased by 0.006±0.001, 0.019±0.002, and 0.013±0.001 Abs/min, respectively. The paired sample t-test results discovered that the activity of AChE, GST, and oxidase increased significantly (p<0.05) (Table-3).

**Table 3:** AChE, GST, and oxidase activity in head lice after *in vitro* treatment of I% permethrin and 6-paradol

Treatment	n	Enzyme	Mean±SD		Paired-samples t-test	
	
			T0	T5 or T10	t-value	sig. (two-tailed)
Permethrin 1%	27	AChE	0.201±0.004	0.262±0.006	19.673	0.000
		GST	0.310±0.007	0.501±0.024	19.686	0.000
		Oxidase	0.928±0.007	1.062±0.007	8.496	0.000
6-paradol						
0.00005%	30	AChE	0.225±0.058	0.401±0.023	14.075	0.000
		GST	0.391±0.053	0.201±0.004	21.678	0.000
		Oxidase	0.966±0.345	1.003±0.345	6.566	0.000
0.0001%	30	AChE	0.245±0.018	0.323±0.028	20.245	0.000
		GST	0.415±0.037	0.850±0.111	22.662	0.000
		Oxidase	1.308±0.235	1.385±0.163	2.348	0.022
0.00015%	27	AChE	0.286±0.068	0.390±0.099	18.591	0.000
		GST	0.449±0.087	0.907±0.187	21.095	0.000
		Oxidase	2.446±0.498	2.492±0.517	16.253	0.000

T0=0 min, T5=5 min, T10=10 min. AChE (0 min and 10 min), GST (0 min and 5 min). Oxidase (0 min and 5 min), sig (significant). AChE=Acetylcholinesterase, GST=Glutathione S-transferase

In the 6-paradol group, all head lice (100%, 87/87) indicated increased values of Abs AChE (0-10 min), GST (0-5 min), and oxidase (0-5 min) (Figure-5). In the 6-paradol treatment with a concentration of 0.00005%, there was an increase in the mean Abs value of AChE by 70.6±29.49%, GST by 97.9±9.4%, and oxidase by 2.8±3.06%. At 0.0001%, the mean increase in Abs values from AChE was 31.9±2.28%, GST was 104.0±9.65%, and oxidase was 7.4±8.4%. At 0.00015%, the mean increase in Abs values of AChE, GST, and oxidase increased by 34.5±7.77%, 98.0±19.08%, and 1.9±0.31%, respectively (Table-3).

Similar to the permethrin group, in the 6-paradol group, fluctuating increases in AChE, GST, and oxidase activity were found in each individual head lice (Figure-6). For example, in the 6-paradol treatment with a concentration of 0.00005%, the average increase in AChE enzyme activity (Abs/min) was 0.017±0.004 Abs/min, GST was 0.039±0.009 Abs/min, and oxidase was 0.008±0.001 Abs/min. At 0.0001%, the average increase in the activity of AChE, GST, and oxidase enzymes increased by 0.008±0.001, 0.044±0.07, and 0.008±0.007 Abs/min, respectively. In addition, at 0.0015%, the average increase in the activity of AChE, GST, and oxidase enzymes increased by 0.010±0.003 Abs/min, 0.046±0.010 Abs/min, and 0.005±0.001 Abs/min, respectively (Table-3).

The paired sample t-test results showed that the 6-paradol group at concentrations of 0.00005%, 0.0001%, and 0.00015% showed that all AChE, GST, and oxidase activities increased significantly (p<0.05) (Table-3).

### Electron microscopic changes in lice

**Figure-7:**
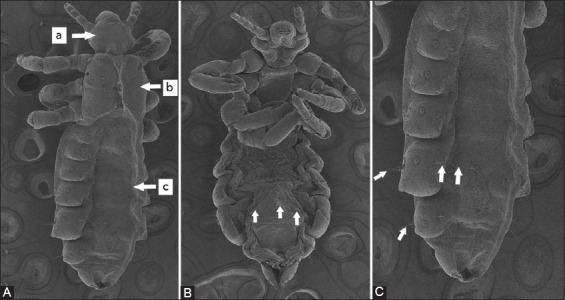
(A-C)Ultrastructural changes of head lice after *in vitro* treatment with permethrin. There are obvious changes in the abdomen, especially sensory hair loss (white arrows) in b and c. Head (a), thorax (b), and abdomen (c).

Figure-7 shows SEM images of the lice treated with permethrin. After *in vitro* treatment with permethrin, smooth outer architecture, head (antennae and eyes), thorax (legs with hook-like claws), and abdomen (respiratory spiracles) did not exhibit any ultrastructural morphological changes. Permethrin caused the loss of some sensory hairs of the dead lice.

**Figure-8:**
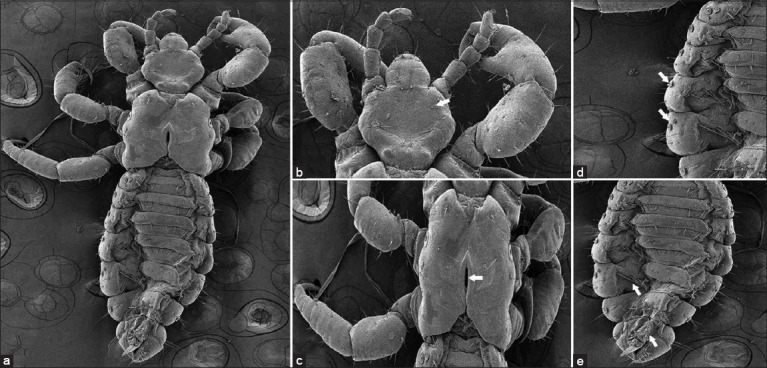
Ultrastructural changes of head lice after *in vitro* treatment with 6-paradol. The whole body of head lice (a). Obvious ultrastructural changes in head lice (white arrows) were found on the head (b), spiracle (c), thorax (d), and abdomen (e).

Figure-8 shows SEM images of the lice treated with 0.00015% 6-paradol. This study used the dead lice exposed to 0.00015% of 6-paradol in 60 min to represent SEM changes for the entire experiment. After *in vitro* treatment with 6-paradol (0.00015%), smooth outer architecture, the head, thorax, and abdomen exhibited no ultrastructural morphological changes in the lice. These findings showed that 6-paradol damaged the chitin layers of the head and thorax, causing an irregularly or abnormally shaped head and thorax. Furthermore, the respiratory spiracles and chitin layers of the abdomen were damaged, while some sensory hairs fell out.

## Discussion

This study demonstrated the low efficacy of permethrin (1%) against head lice using *in vitro* experiments (36.7% mortality, 11/30). During a 60-min period, permethrin killed lice slowly, with LT_50_ and LT_90_ values of 37.666 and 354.670 min, respectively. The low efficacy of permethrin indicated that lice from East Jakarta were resistant to this specific pediculicide. This was demonstrated by 63.3% (19/30) of the lice treated with permethrin survived 60 min of exposure. This study’s determination of permethrin-resistant head lice was based on the parasitolo- gical approach, an *in vitro* bioassay method. Although 6-paradol killed lice quicker than permethrin, its efficacy was low. To the best of our knowledge, this study is the first to report that lice from East Jakarta show resistance to permethrin and 6-paradol.

Similarly, Kristensen *et al*. [33] in Denmark used an *in vitro* bioassay approach to determine the resistance of head lice to permethrin; 17 of 24 samples (Danish head lice population) were examined for permethrin resistance, and all of the lice survived different doses for 4 h. Six samples had between 3% and 25% morta- lity rates, and one sample showed 60% mortality. Many parasitological surveys to determine the resistance of lice to permethrin have been reported in Europe, precisely the Czech Republic [34], the UK [35], and Denmark [33] and Israel [36], the USA [37], Argentina [38], Japan [39], and Australia [40]. Results from the study are in agreement with those from a study by Kristensen *et al*. [33], who found that head lice were permethrin-resistant using an *in vitro* bioassay method.

Clinical trials of permethrin have not been conducted among residents in Jakarta to determine its efficacy. No studies have examined *kdr*-type gene mutations related to the failure of permethrin to treat head lice. One previous study reported that the efficacy of permethrin was poor to fair [1]. Recently, Kalari *et al*. [41] reported that the efficacy of permethrin for treating lice in Iran ranged from 60% to 80%. Heukelbach *et al*. [42] indicated that the efficacy of dimethicone for treating lice was 98%. *kdr*-type gene mutations cause the poor efficacy of permethrin. Three *kdr*-type point mutations (M815l, T917l, and L920F) in the VGSC α-subunit gene are associated with permethrin-resistant phenotypes responsible for *kdr*-type resistance [13-15]. These *kdr* mutant alleles have been found worldwide [10], and they are most prevalent in countries with easy access to pediculicidal agents [12]. In Germany, Bialek *et al*. [43] reported that *kdr* mutant alleles were not associated with clinical treatment failure of pyrethroids. In Senegal, it was reported that the presence of a point mutation of GluCI is associated with ivermectin-resistant head lice [17]. In addition, several novel *kdr* point mutations of the VGSC α-subunit gene have been discovered. For example, in Iran, six-point mutations, H813P (located in IIS1-2 extracellular loop), I927F, L928A, R929V, L930M, and L932M (located in IIS5), were found in head lice resistant to pyrethroid insecticides [18] and in Mexico, a new point mutation, T929I, was found in head lice resistant to insecticides, especially pyrethroids [19].

Our study showed that the resistance of lice to permethrin is mediated by detoxifying enzyme activity. In the permethrin group, AChE, GST, and oxidase activities increased by 30.6%, 61.7%, and 14.5%, respectively. Statistically, the activity of these enzymes increased significantly. Abs values of these enzymes were also increased in the 6-paradol group. These results agree with those from González Audino *et al*. [31], who reported that increased monooxygenase, esterase, and GST activity were associated with resistance of lice to permethrin. Furthermore, Brogdon [22] reported that elevated detoxifying enzyme activity levels (such as AChE, GST, esterase, and oxidase) were associated with insecticide-resistant species of *Anopheles*. Thus, it can be deduced that the mechanism of permethrin-resistant lice is likely associated with increased detoxifying enzyme activity.

Our study used 6-paradol, a bioactive plant compound, to compare permethrin’s efficacy (1%) because synthetic pediculicides, such as carboxyl, DDT, lindane, and malathion, have also been reported to cause resistance [10]. The compound 6-paradol is a non-pungent metabolite of shogaol [25]. A previous study indicated that 6-paradol exhibits neuroprotective effects to decrease neuroinflammation-associated central nervous system disorders [25,44]. Kim *et al*. [45] reported that 6-paradol inhibited cytochrome P450 enzyme activity in human liver microsomes. These findings showed that 6-paradol was associated with increased AChE, GST, and oxidase activity. This study also showed significantly elevated oxidase activity in the 6-paradol group (Table-3).

Plant products can be used as safe alternatives to pediculicidal agents due to low toxicity to mammals and easy biodegradability [46]. Citronella java (essential oils) from *Cymbopogon nardus* (0.25-mg/cm^2^) exihibited adulticidal activity against female *P. h. capitis* [47]. Spearmint, clove, thyme, eucalyptus, and anise essential oils, as potential pediculicides, showed different levels of effectiveness against head lice [48]. Akkad *et al*. [49] reported that after 1 h of exposure *in vitro*, 1% ivermectin and lemon juice caused 100% mortality in lice; tea tree oil caused 96.7% mortality, and virgin olive oil caused 23.3% mortality. Candy *et al*. [50] reported that clove oil diluted in coconut oil or sunflower oil showed adulticidal activity (>90% lice mortality after 2 h). In our study, 6-paradol exhibited 86.7% mortality after 1 h. Moreover, the effectiveness of 6-paradol was lower than that of ivermectin, lemon juice, and tea tree oils as previously reported by Akkad *et al*. [49]

Electron microscopic findings indicated that permethrin did not cause damage to the head, thorax, or abdomen of the lice (Figure-7), while 6-paradol seriously damaged the head, thorax, respiratory spiracles, and abdomens of the dead lice. The 6-Paradol caused damage to the chitin layer of the head, thorax, and abdomen so that the shape of the head, thorax, and abdomen was abnormal (Figure-8). Akkad *et al*. [49] reported that olive oil, tea tree oil, lemon juice, and ivermectin caused electron microscopic alterations in dead-head lice; they damaged sensory hairs, respiratory spiracles, and/or clinching claws. Electron microscopic alterations are caused by antioxidant stress induced by pediculicidal agents, ivermectin, and essential oils. Thus, the mechanism by which pediculicides from chemical and natural products kill human head lice can be visualized using electron microscopic changes.

## Conclusion

Our findings showed that human head lice, *P. h. capitis*, from East Jakarta, were permethrin-resistant and 6-paradol treatment with these pediculicides yielded increases in AChE, GST, and oxidase activity. The electron microscopic findings indicated that permethrin did not induce damage to the head, thorax, and abdomen of the lice. In contrast, 6-paradol severely damaged the dead lice’s head, thorax, respiratory spiracles, and abdomen. Alternative pediculicidal agents are urgently required to create optimal management strategies.

## Authors’ Contributions

RS, LS: Designed the study. RS, RA, APA, and NSL: Collected the samples from the fields and conducted bioassay. RS, RA, APA, and YY: Conducted the biochemical assay. RS and RA: Examined head lice with scanning electron microscope. RS, LS, RW, IPS, and YY: Carried out data analysis. RS, LS, RA, APA, YY, RW, NSL, and IPS: Drafted the manuscript. All authors read and approved the final manuscript.
